# Dietary emulsifiers consumption alters anxiety-like and social-related behaviors in mice in a sex-dependent manner

**DOI:** 10.1038/s41598-018-36890-3

**Published:** 2019-01-17

**Authors:** Mary K. Holder, Nicole V. Peters, Jack Whylings, Christopher T. Fields, Andrew T. Gewirtz, Benoit Chassaing, Geert J. de Vries

**Affiliations:** 1 Neuroscience Institute, Georgia State University, Atlanta, GA 30303 USA; 2 Institute for Biomedical Sciences, Georgia State University, Atlanta, GA 30303 USA; 3 Present Address: School of Psychology, Georgia Institute of Technology, Atlanta, GA 30332 USA

## Abstract

Dietary emulsifiers carboxylmethylcellulose (CMC) and polysorbate 80 (P80) alter the composition of the intestinal microbiota and induce chronic low-grade inflammation, ultimately leading to metabolic dysregulations in mice. As both gut microbiota and intestinal health can influence social and anxiety-like behaviors, we investigated whether emulsifier consumption would detrimentally influence behavior. We confirmed that emulsifier exposure induced chronic intestinal inflammation, increased adiposity, and altered gut microbiota composition in both male and female mice, although the specific microboal taxa altered following emulsifier consumption occurred in a sex-dependent manner. Importantly, emulsifier treatment altered anxiety-like behaviors in males and reduced social behavior in females. It also changed expression of neuropeptides implicated in the modulation of feeding as well as social and anxiety-related behaviors. Multivariate analyses revealed that CMC and P80 produced distinct clustering of physiological, neural, and behavioral effects in male and female mice, suggesting that emulsifier treatment leads to a syndrome of sex-dependent changes in microbiota, physiology, and behavior. This study reveals that these commonly used food additives may potentially negatively impact anxiety-related and social behaviors and may do so *via* different mechanisms in males and females.

## Introduction

The notion that the viscera or gut influences our emotions dates back over 100 years^1^, and recent studies suggest this influence may be related to pathology. Indeed, a high comorbidity exists between gastrointestinal and psychiatric illnesses^2–4^. An emerging focus of the gut-brain axis is the intestinal microbiota, the large and diverse community of microbes that reside in the gut, which has been shown to influence anxiety-like and social behaviors in mice^5,6^. For example, mice reared in the absence of microbiota (germ-free mice) show lower anxiety-like behavior than conventionally-colonized mice^7–9^, and introducing microbiota around the time of weaning partially normalizes anxiety-like behaviors^7,8,10,11^. In addition, germ-free mice show reductions in social behavior^12^, and early life exposure to antibiotics also affects anxiety-like and social behaviors^13^. Oral exposure to pathogenic bacteria increases the number of pro-inflammatory bacteria strains in the gut, and gastrointestinal inflammation increases anxiety- and depression-like behaviors in mice^14–18^. In contrast, some probiotics with anti-inflammatory properties^19^ reduce such behaviors^5,14,20–22^, and prebiotics, which can act as food sources for the generation of generally beneficial short-chain fatty acids (SCFA), can reduce anxiety- and depression-like behaviors in mice^23^.

One potential mechanism for influencing intestinal microbiota, and thereby the inflammatory state, is through diet. The Western diet is high in sugar, fats, red meats, refined grains, and processed foods containing food additives for both preservation and flavor and/or texture enhancement^24,25^. Adding carboxymethylcellulose (CMC) or polysorbate-80 (P80), commonly used emulsifying food additives, to the diet induces low-grade inflammation, obesity, and metabolic abnormalities in mice^26,27^. The same treatments also promote microbial encroachment within the intestinal mucus barrier and alter microbiota species composition toward a more pro-inflammatory potential. Germ-free animals are protected from intestinal inflammation and metabolic abnormalities following emulsifier exposure, and transplant of microbiota from emulsifier-treated animals to germ-free recipient mice is sufficient to confer metabolic alterations, indicating that microbiota drive this phenotype^26^. Taken together, these data further support the concept that microbiota composition is important for health, and that perturbations of the intestinal microbiota by modern stressors, such as emulsifiers, can lead to aberrant physiology.

In the present study, we examined the effects of emulsifier consumption on brain and behavior. We found that emulsifier treatment altered anxiety-like and social behaviors, and did so in a sex-specific manner. Such sex-specific differences were paralleled by emulsifiers differentially impacting microbiota composition, inflammation, and metabolism. These results demonstrate the potential for food additives that alter the intestinal environment to broadly influence physiology and behavior.

## Results

### Effects of emulsifiers on host physiology and metabolism

**Figure 1:**
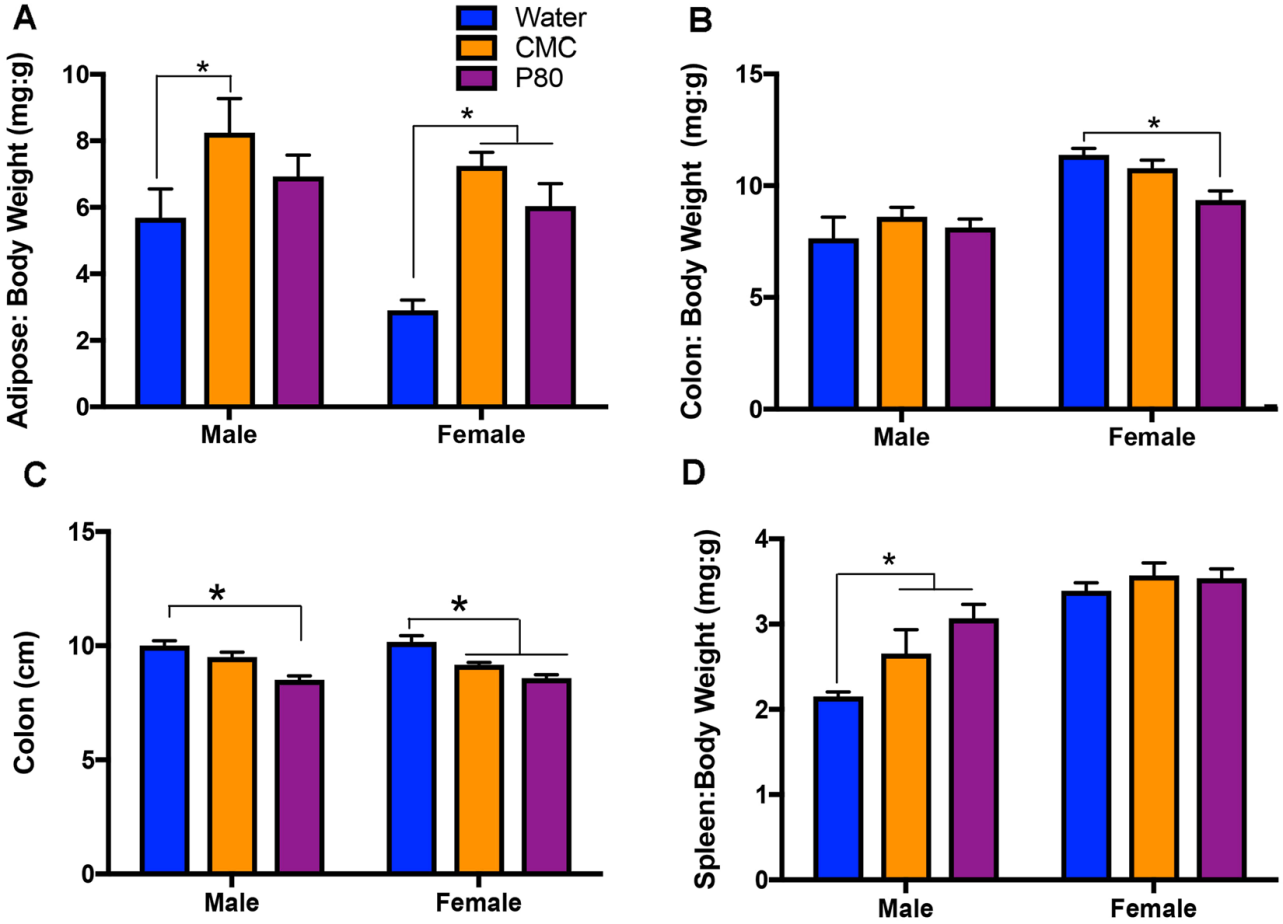
Dietary emulsifiers promote physiological changes consistent with metabolic syndrome. Male and female C57Bl/6 mice were exposed to drinking water containing CMC or P80 (1%) for 12 weeks. (**A**) There was a main effect of treatment with emulsifiers on fat-pad mass [F_(2,29)_ = 12.48, p < 0.001]. There is also a main effect of sex on adiposity such that males had greater fat mass than did females F_(1,29)_ = 7.65, p < 0.01]. Post-hoc comparisons indicate that both CMC and P80 increased fat mass in females, but in males only CMC treatment increased fat mass compared to respective water-treated controls (*p < 0.05). (**B**) There was a significant interaction of emulsifier treatment and sex on colon weights [F_(2,29)_ = 3.383, p < 0.05], with post-hoc comparisons indicating that P80 treatment significantly reduced colon weights in female compared to water-treated controls (*p < 0.05). (**C**) Emulsifier treatment also had a significant main effect on colon lengths in male and female mice [F_(2,29)_ = 28.70, p < 0.0001] such that females treated with both emulsifiers and males treated with P80 had significantly shorter colons compared to their respective water-treated controls (*p < 0.05). (**D**) There was a significant main effect of both emulsifier consumption [F_(2,29)_ = 5.312, p < 0.05] and sex on spleen weights [F_(1,29)_ = 43.31, p < 0.0001]. Post-hoc comparisons indicate that treatment with both CMC and P80 increased spleen weight compared to water-controls in males, but not females (*p < 0.05). Data are represented as means + SEM (n = 5–6).

In accord with our previous work^26^, twelve weeks of exposure to emulsifiers carboxymethylcellulose (CMC) or polysorbate (P80) *via* drinking water led to a marked increase in abdominal adiposity that was associated with chronic mild intestinal inflammation, as revealed by shorter colons and increased spleen weight (Fig. 1 and Supplemental Fig. 1).

### Impact of emulsifier consumption on fecal microbiota composition

**Figure 2:**
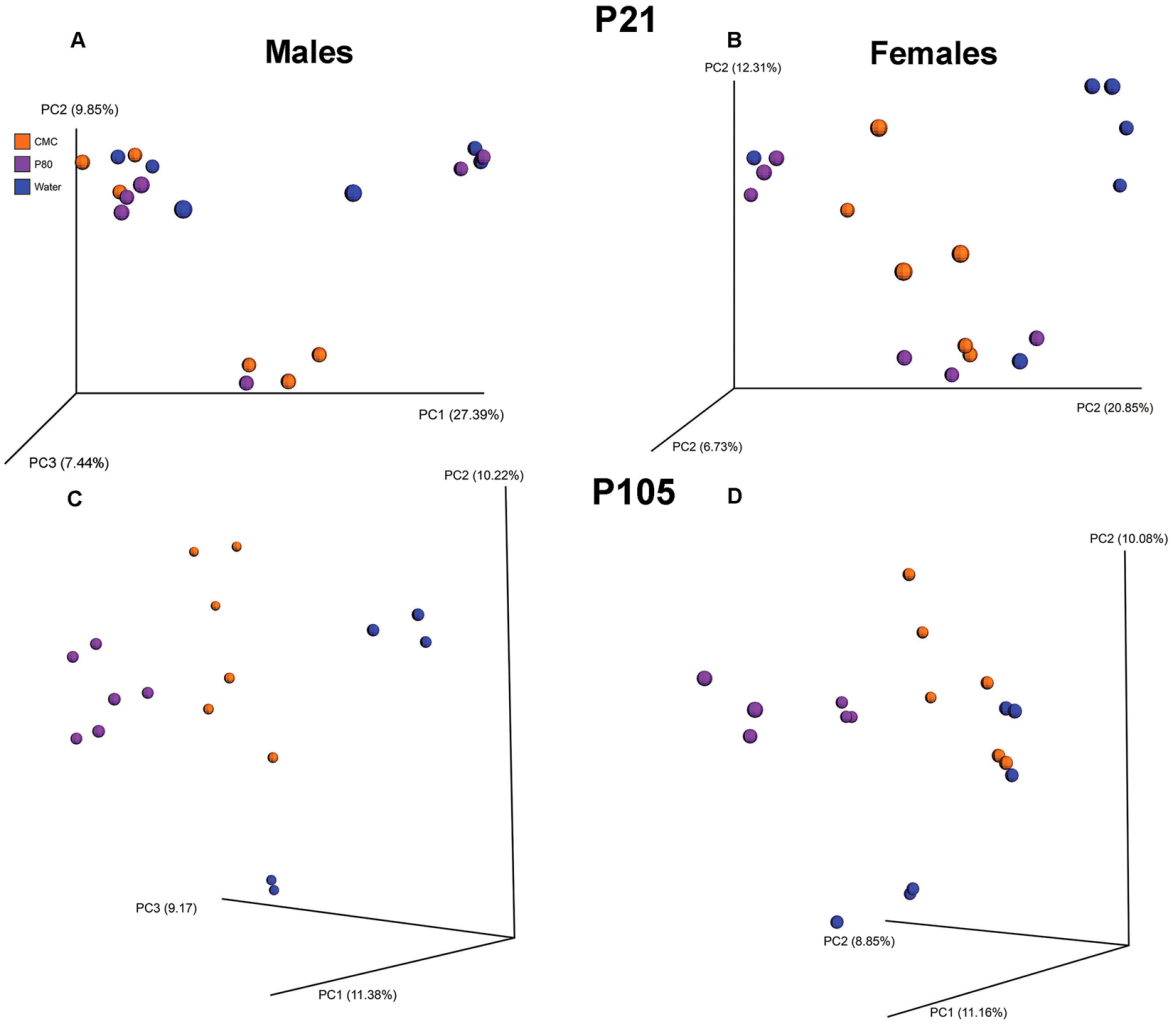
Effects of dietary emulsifiers on microbiota. Principal coordinates analysis (PCoA) of the unweighted UniFrac distance matrix of fecal microbiota in male (**A**,**C**) and female (**B**,**D**) mice at the time of weaning, P21 (**A**,**B**) and at the time of collections, P105 (**C**,**D**). Treatment of the mouse is indicated by point color (blue, water; orange, CMC; purple, P80).

We next used 16S rRNA sequencing to determine the effects of emulsifier consumption on microbiota composition. Using Principal Coordinate Analysis (PCoA) of the unweighted UniFrac distances, we first examined the differences in microbiota composition before treatments begin (P21). As expected, prior to any treatment, the microbiota did not differentially cluster prior to treatment (Fig. 2A,B). In addition, LefSe analyses identified very few operational taxonomic units (OTUs) with an altered abundance between treatment (Supplemental Fig. 2A,B,E,F). Importantly, following twelve weeks of emulsifier exposure (P105), male and female animals harbored distinct microbiota composition based on treatment (Fig. 2C,D, Permanova < 0.001). LefSe analysis conducted in males and females separately indicated that several taxa differ based on treatment: in males, emulsifier comsumption reduced the abundance of the Firmicutes phylum and *Oscillospria*, *Coprococcus*, and *rc4_4* genera (Supplemental Fig. 2C,D). CMC-treated males exhibited higher abundance of the genus *Dorea* whereas P80-treatment increased the abundance of the genera *Bacteroides*, *Burkholderia*, *Clostridium*, and *Veillonella*. In females, emulsifier treatment reduced abundance of *Bacteroides*, *Sphingomondales*, *Sphingomonas*, *and Ruminococcus* (Supplemental Fig. 2G,H). CMC-treated females showed increases in *Anaeroplasma*; whereas P80 treatment increased the relative abundance of the Proteobacteria phylum and of *Clostridium* and *Burkholderia* genus.

**Figure 3:**
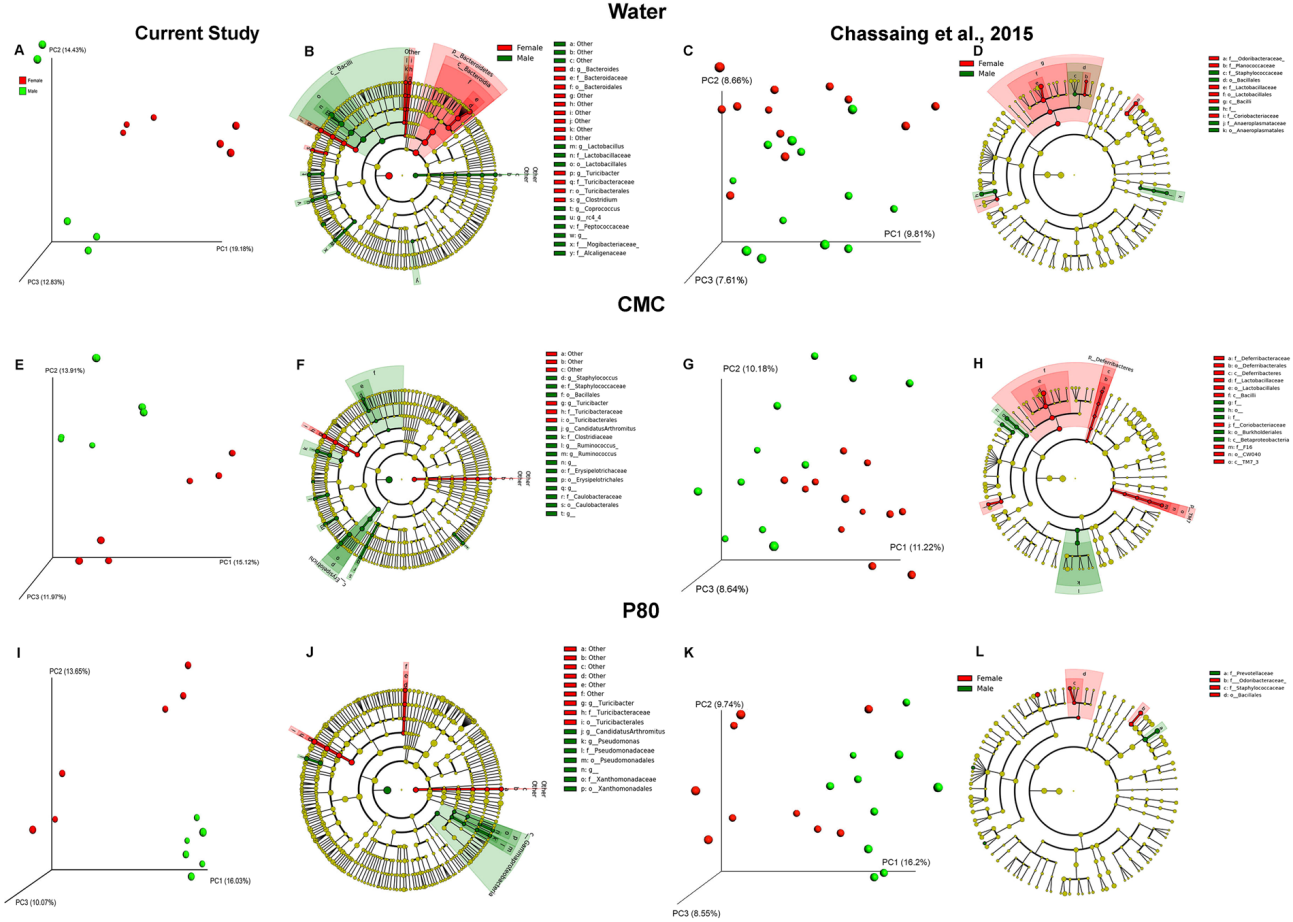
Sex differences in microbiota in mice treated with dietary emulsifiers. Male and female C57Bl/6 mice were exposed to drinking water containing CMC or P80 (1%) in the present study and in data previously reported in Extended Data Fig. 3 in Chassaing *et al*.^26^. Principal coordinates analysis (PCoA) of the unweighted UniFrac distance matrix of fecal microbiota showing clustering by sex in (**A**,**C**) water-, (**E**,**G**) CMC- and (**I**,**K**) P80- treated mice. Sex of the mice is indicated by point color (red, female; green, male). Linear discriminant analysis coupled with Effect Size (LEfSe) was used to identify taxa that differ significantly between male and female mice within water, CMC, and P80 treatments.

We next analyzed microbiota composition in animals from the current study and in animals from our previous work^26^ (Fig. 3 and Supplemental Fig. 3) in order to examine sex differences in the microbial community structure. Such analysis revealed an impact of sex on microbiota composition, for each experimental group (water, CMC and P80, Fig. 3). When each treatment group was examined separately, there were sex differences in community composition. (Fig. 3). For example, within the water-treated controls, bacteria from genera *Bacteroides* and *Clostridium* were more abundant in females, whereas bacteria within the genera *Lactobacillus* and *Coprococcus* were more highly present in males. (Fig. 3B,D).

Treatment with emulsifiers changed the gut microbiota of males and females differently (Fig. 3). For example, the sex differences in the genera *Bacteroides*, *Closteridium*, *Lactobacillus* and *Coprococcus* are eliminated following CMC treatment. Some new sex differences also emerged: following CMC treatment in males, an increased abundance in bacteria pertaining to the genera *Staphylococcus* and *Ruminococcus* (Fig. 3F) was observed, and females harbored more bacteria within the phyla *Deferribacteres* and *TM7* (Fig. 3H). Following P80 treatment, males displayed an increased abundance of the genus *Pseudomonas* (Fig. 3J,L).

### Effects of emulsifiers on behavior

#### Anxiety-like behavior - Open Field Test

**Figure 4:**
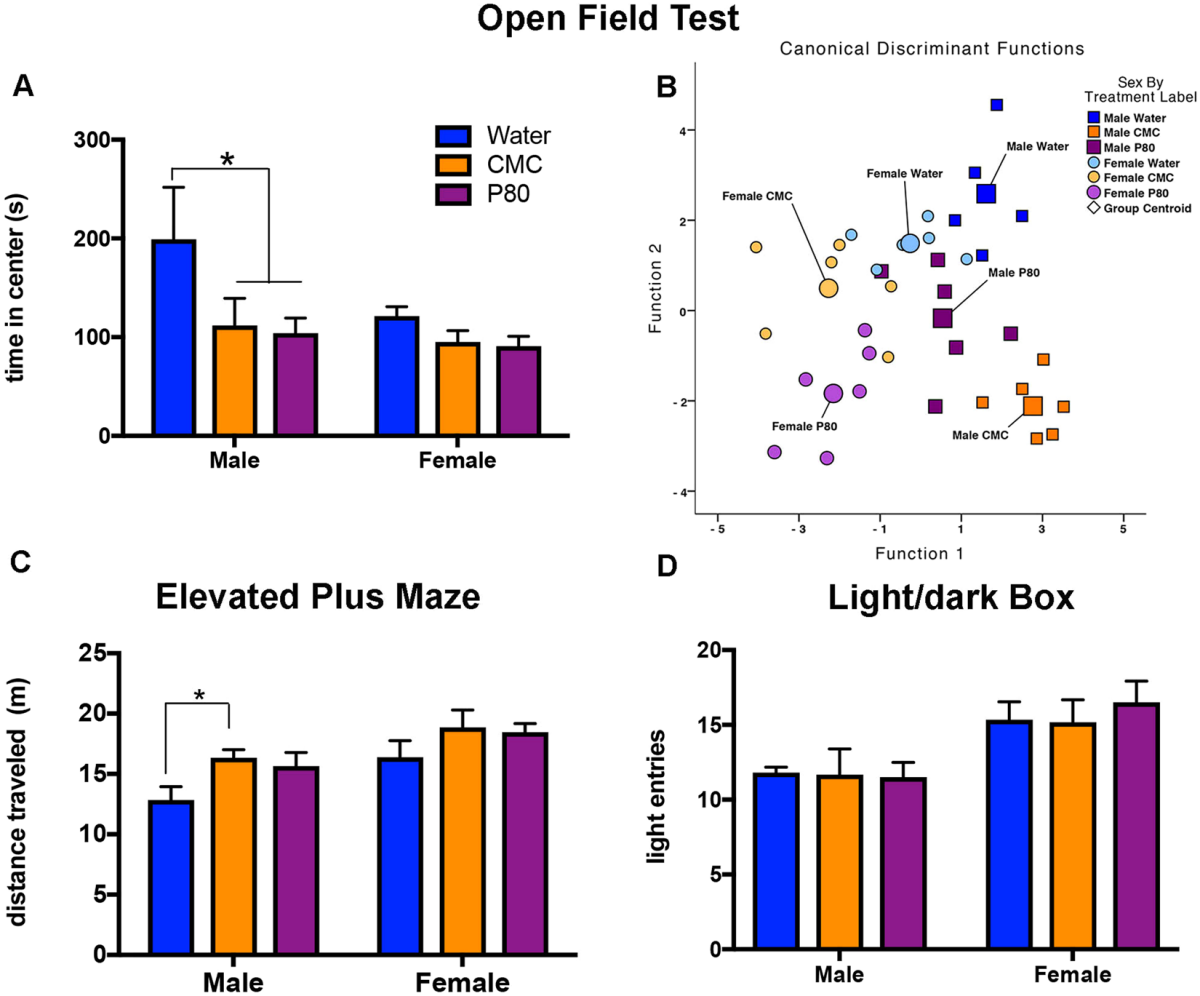
Dietary emulsifiers alter anxiety-like behaviors in male and female mice. (**A**) There was a main effect of emulsifier treatment to decrease the time spent in the center of the open field test [F_(2,29)_ = 4.14, p < 0.05]. Post-hoc analyses indicate that in males, treatment with emulsifiers decreases in the time spent in the center, compared to water-treated controls (*p < 0.05). (**B**) Multivariate test statistics measured the impact of additional behavioral measures captured in the automated open field apparatus (Table 1). The canonical discrimination function plot and Wilk’s lambda revealed a significant separation of groups by sex and emulsifier consumption along five discriminant functions [*Λ* = 0.010, *Χ*^2^(30) = 108.204, p < 0.01]. (**C**) Emulsifier consumption increased the total distance traveled in the elevated plus maze [F_(2,29)_ = 3.94, p < 0.05]. Post-hoc analyses indicate that in males, treatment with CMC significantly increased the distance traveled in the EPM (*p < 0.05). In addition, there was a main effect of sex [F_(1,29)_ = 10.42 p < 0.01], such that females traveled a greater distance, compared to males, regardless of treatment. (**D**) There was no significant effect of emulsifier treatment on the total number of entries into the light portions of the light/dark box. Irrespective of treatment, however, female mice made significantly more entries into the light portion of the light/dark box [F_(1,29)_ = 13.76, p < 0.001]. Data are represented as means + SEM (n = 5–6).

We next sought to determine the impact that emulsifier consumption and associated alterations in microbiota composition might have on behavior. We observed that, in male animals, emulsifier treatment reduced the time spent in the center portion of the open field (Fig. 4A) without affecting the total distance traveled in the apparatus (Supplemental Fig. 4A). In addition, there was a trend towards a main effect of sex, such that females spent less time in the center, compared to males (p = 0.07; Fig. 4A), mostly driven by the time spent in the center in the male water group. Multivariate test statistics revealed that the behaviors in the open field test separated significantly by sex and emulsifier treatment along five discriminant functions (Table 1). Function 1 explained 43.9% of the variance in the data set (*R* = 0.898) and the number of stereotypic beam breaks maps most highly onto this function (r = 0.383). The canonical discriminant function plot reveals the effects of each individual emulsifier treatment on each of the sexes for these two functions in the open field behaviors. Emulsifier consumption causes a separation of the aggregate open field behaviors from the water-treated controls. Moreover, the changes in the open field behaviors are similar in P80-treated male and female mice, whereas, CMC may exert unique effects in male and females (Fig. 4B).

**Table 1:** Structure Matrix of Discriminant Analysis for Open Field Behavior.

Measured Outcomes	Function				
	1	2	3	4	5
Circling Counts	0.383*	0.118	−0.119	0.053	0.153
Resting Time (sec)	0.298*	0.186	−0.023	0.160	0.214
Time in Center Zone (sec)	0.147*	−0.109	−0.104	0.145	−0.091
Number of Entries into Center Zone	0.155	0.262*	0.051	0.260	0.159
Jump Counts	−0.259	0.213	0.435*	0.138	−0.048
Average Velocity	−0.295	0.150	0.304*	0.190	−0.164
Counter Clockwise Reversals	0.057	−0.127	0.372	0.381*	−0.039
Ambulatory Episodes	0.080	0.280	−0.071	0.304*	−0.021
Vertical Time (sec)	0.062	−0.060	−0.159	0.256*	0.141
Jump Time (sec)	−0.007	0.235	0.112	0.043	0.450*
Circling Time (sec)	−0.210	−0.066	0.205	0.022	0.406*
Ambulatory Time (sec)	−0.144	−0.090	0.181	0.057	0.401*
Ambulatory Counts	−0.179	−0.127	0.202	−0.003	0.389*
Ambulatory Distance (cm)	−0.115	−0.053	0.165	−0.005	0.345*
Clockwise Reversals	−0.032	0.242	0.087	−0.179	0.317*

Pooled within-group correlations between discriminating variables and standardized canonical discriminant functions. The variables are ordered by absolute size of correlation within each the functions (*indicates the largest absolute correlation between each variable and any discriminant function).

#### Anxiety-like behavior - Elevated Plus Maze Test

Treatment with emulsifiers did not affect time spent in, nor number of entries into, either the open or closed arms in the elevated plus maze test (Supplemental Fig. 4). Emulsifier consumption did, however, increase the distance travelled in this behavioral test (Fig. 4C), suggesting that although emulsifier consumption did not increase anxiety in this test, it impacted locomotor behavior.

#### Anxiety-like behavior - Light/Dark Box

Treatment with emulsifiers did not affect time spent in the light nor the number of entries into the light (Fig. 4D and Supplemental Fig. 4F), suggesting that emulsifiers did not impact anxiety in the light/dark box test. Irrespective of treatment, female mice made significantly more entries into the light, but the total amount of time spent in the light did not differ from male mice (Fig. 4D).

#### Anxiety-like behavior - Marble Burying Task

Treatment with emulsifiers did not significantly affect the number of marbles buried (p = 0.91; data not shown) or the latency to bury the first marble (p = 0.69; data not shown).

#### Sociability – Three chambers test

**Figure 5:**
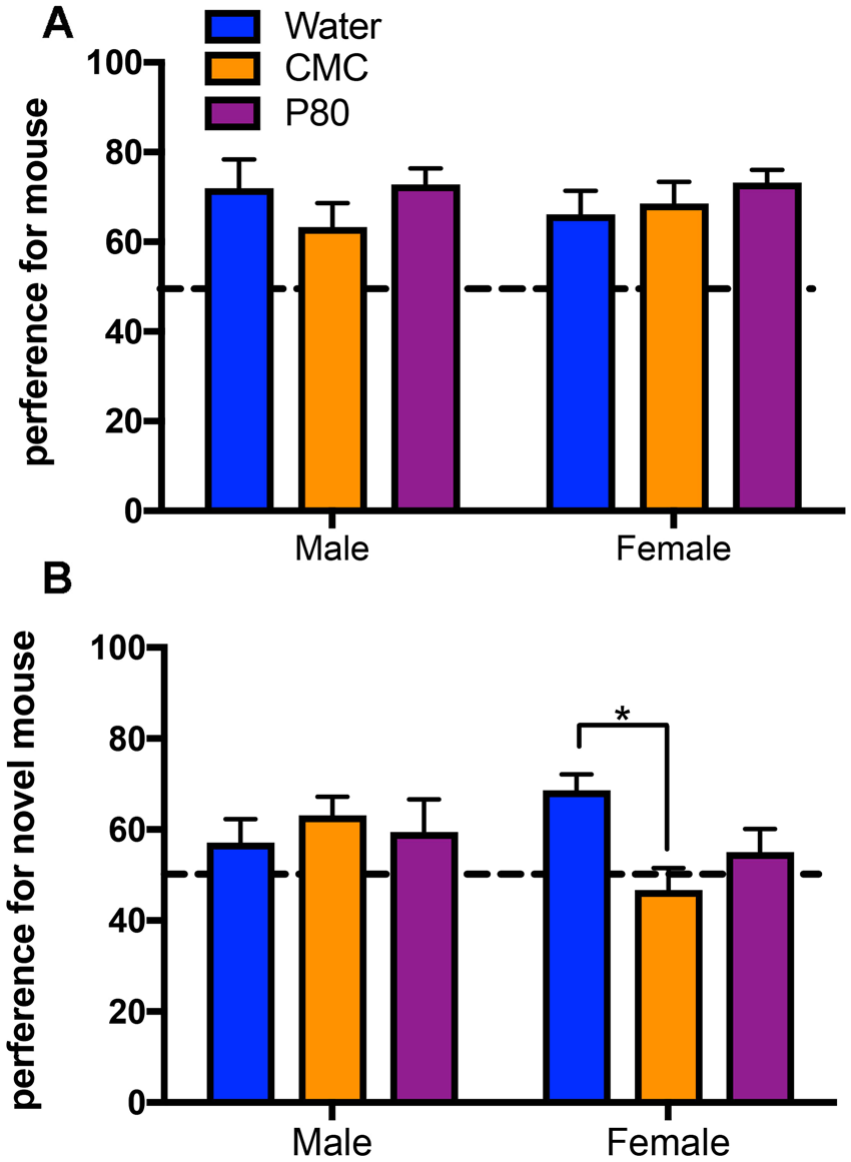
Dietary emulsifiers decrease preference for social novelty in female mice. (**A**) There was no significant main effect of emulsifier consumption [F_(2,28)_ = 1.08, p = 0.35] or sex [F_(1,28)_ = 0.00003 p = 0.99] on the preference for investigating a novel, conspecific mouse during the sociability test in the three-chambered sociability apparatus. In addition, there was no sex by treatment interaction on this measure [F_(2,28)_ = 0.67, p = 0.52] (**B**). Emulsifier treatment and sex interacted on the preference for investigating a second novel, conspecific mouse during the preference for social novelty test in the three-chambered sociability apparatus [F_(2,29)_ = 3.71, p < 0.05]. In addition, post-hoc comparisons indicate that treatment with CMC significantly decreased the preferences of female mice for the novel mouse (*p < 0.05). Data are represented as means + SEM (n = 5–6).

Treatment with emulsifiers did not affect social interaction as measured by the percent of time spent investigating a novel mouse when given the choice to investigate that mouse or a novel object (Fig. 5A). However, if given a choice between a novel or a familiar mouse, emulsifier treatment lowered the preference for the novel mouse compared to water-treatment in females animals (Fig. 5B). Indeed, post-hoc comparisons indicated that CMC consumption in female mice significantly reduced the preference for the novel mouse. In addition, there was a strong trend towards a reduced preference for the novel mouse following P80 consumption in female mice (p = 0.06) (Fig. 5).

#### Depression-like behavior - Forced Swim Test

Treatment with emulsifiers did not affect the duration of immobility (p = 0.92; data not shown) or the latency to first bout of immobility in the force swim test (p = 0.30; data not shown).

### Effects of emulsifiers on neural correlates

**Figure 6:**
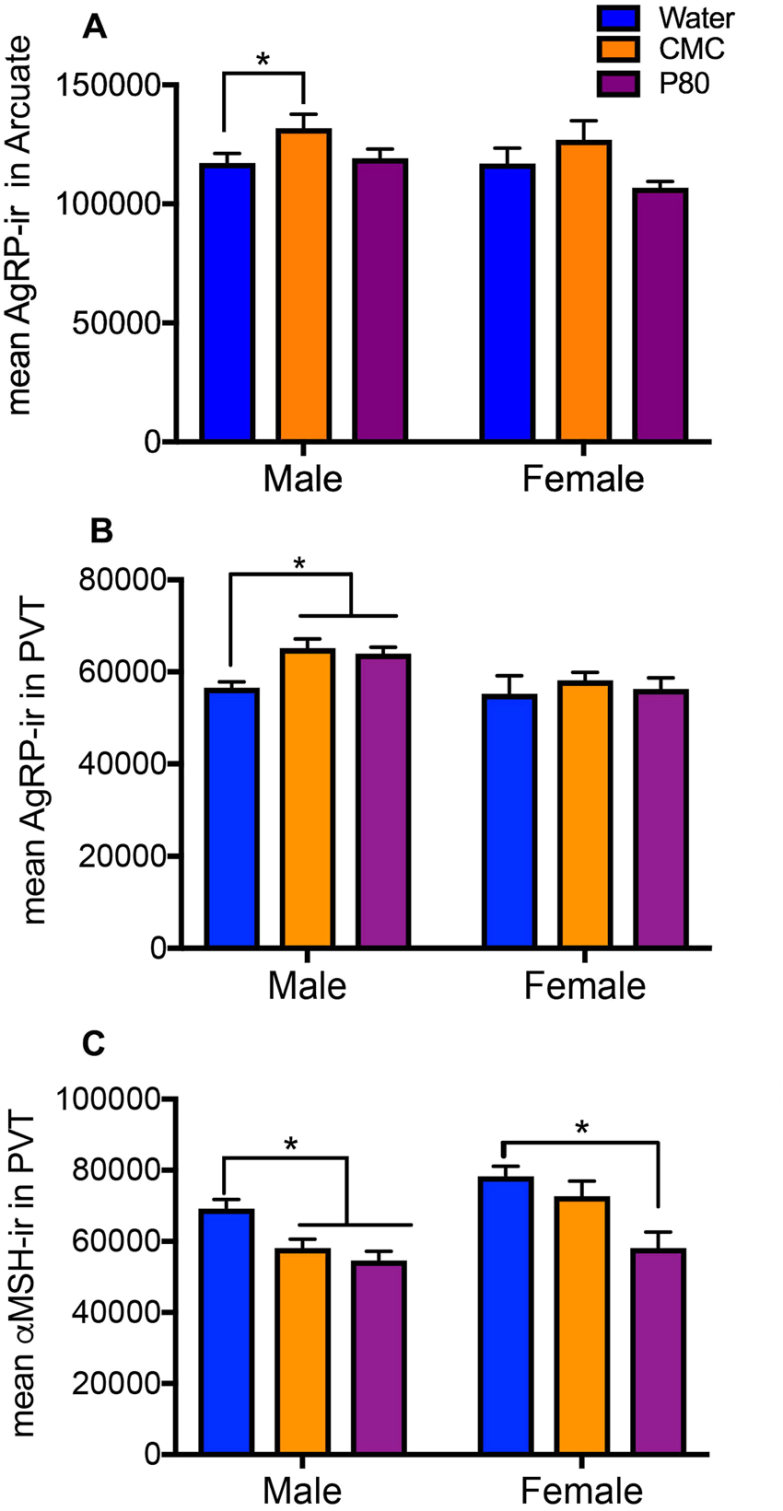
Dietary emulsifiers alter neuropeptide immunoreactivity in male and female mice. (**A**) There was a main effect of emulsifier consumption on agouti-related peptide (AgRP)-immunoreactivity (IR) in the arcuate nucleus in both male and female mice [F_(2,29)_ = 4.689, p < 0.05], driven, in part, by the significant increase following consumption of CMC in males (*p < 0.05). (**B**) There was a main effect of sex on AgRP-IR in the paraventricular nucleus of the thalamus (PVT) [F_(1,29)_ = 7.494, p < 0.05], such that males had more AgRP-IR than females. In addition, post-hoc analysis indicated that consumption of emulsifiers significantly increased AgRP-IR in the PVT of males (*p < 0.05). (**C**) Emulsifiers reduced α-Melanocyte Stimulating Hormone (αMSH)-IR in the PVT in male and female mice [F_(2,29)_ = 12.98, p < 0.001]. In addition, there was also a main effect of sex with females having more αMSH-IR than males [F_(1,29)_ = 14.42, p < 0.001]. Data are represented as means + SEM (n = 5–6).

We next investigated the effects of emulsifiers on neuropeptides that influence feeding and anxiety behaviors. Both agouti-related peptide (AgRP) and α-melanocyte stimulating hormone (α-MSH) regulate appetite, energy homeostasis, and anxiety-like behavior. While AgRP stimulate food intake and reduce anxiety-like behaviors, αMSH is acting in opposition to inhibit food intake and increase anxiety-like behaviors^28–30^. In males, consumption of CMC increased AgRP immunoreactivity (IR) in the arcuate nucleus (Fig. 6A) and both CMC and P80 consumption increased AgRP IR in the paraventricular nucleus of the thalamus of male animals (PVT; p = 0.05, Fig. 6B). Emulsifier treatment reduced α-MSH IR in the PVT of both male and female animals (Fig. 6C). Females also have increased αMSH IR compared to males in both the PVT and the arcuate nucleus (p < 0.0001; data not shown). Treatment with emulsifiers was without effect on αMSH IR in the arcuate nucleus (p = 0.95; data not shown) and the PVN (p = 0.91; data not shown).

That dietary emulsifiers induced chronic intestinal low-grade inflammation led us to examine whether emulsifier altered microglia by examining Iba1-immunoreactivity. Emulsifier treatment did not affect total Iba1-immunoreactivity in the PVT, PVN, Arcuate nucleus, or hippocampus (data not shown), suggesting that emulsifier consumption does not lead to gross changes in microglia.

### Multivariate analysis of emulsifier effects

Next, multivariate tests were used in order to measure the impact of emulsifiers on synergistic changes in the behavioral measures, physiological parameters, and immunoreactivity of neuropeptides in the PVT. When these measures were analyzed in combination (excluding microbiota composition analysis), Wilk’s lambda revealed a significant separation of groups by sex and emulsifier treatment along five discriminant functions. The variables that map most highly onto these functions are listed in Table 2. Functions 1 and 2 explain the majority of variance in the data set (cumulatively, 80.0% of the variance), with Function 1 explaining 53.8% of the variance (*R* = 0.945) and Function 2 explaining 26.2% of the variance (*R* = 0.896).

**Table 2:** Structure Matrix of Discriminant Analysis for Physiological, Behavioral, and Neuropeptidergic Effects of Emulsifier Treatment.

Measured Outcomes	Function				
	1	2	3	4	5
Relative Body Weight	0.418*	−0.088	−0.098	−0.242	0.103
Colon Weight	−0.102*	−0.004	0.072	0.022	0.005
MSH-IR in PVT	−0.113	0.520*	0.209	−0.374	−0.0.013
Fat Pad Weight	0.229	0.231*	−0.111	0.163	0.022
Circling Counts	0.044	0.105	−0.489*	0.027	−0.168
Time in Open Arm	0.011	0.008	0.236*	−0.185	−0.058
Spleen Weight	−0.081	−0.082	0.190*	−0.129	0.059
AgRP-IR in PVT	0.076	−0.264	−0.220	−0.358*	−0.082
Number of Marbles Buried	−0.101	−0.042	0.093	−0.269*	−0.172
Time in Light	0.059	−0.045	0.129	0.265*	−0.253
Social Preference	0.050	0.015	−0.011	0.262*	−0.107
Social Interaction	0.003	0.084	0.147	0.169*	−0.129
AVP-IR in PVT	0.072	−0.065	−0.261	−0.150	0.504*
Time in Center Zone	0.058	0.095	−0.039	0.002	−0.423*
Colon Length	0.004	0.021	0.073	0.046	0.156*

Pooled within-group correlations between discriminating variables and standardized canonical discriminant functions. The variables are ordered by absolute size of correlation within each the functions (*indicates the largest absolute correlation between each variable and any discriminant function).

**Figure 7:**
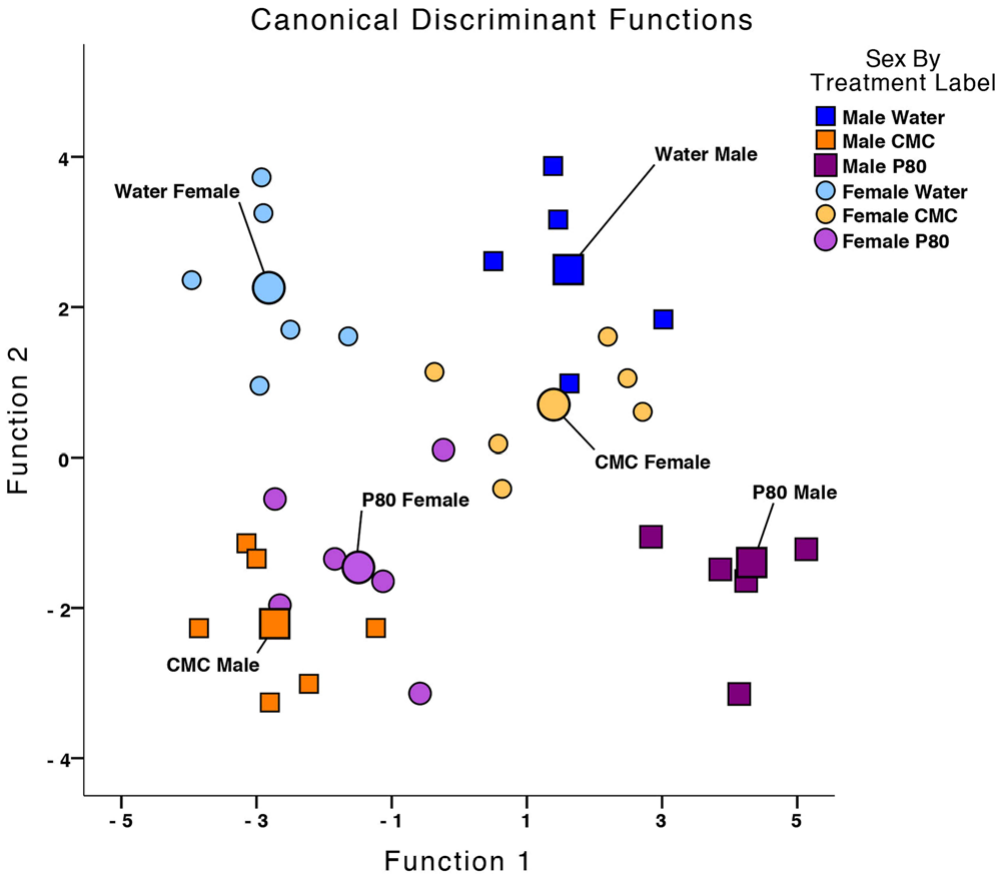
Dietary emulsifiers induce a syndrome of behavioral, physiological, and neural changes. Multivariate analysis of the impact of emulsifiers on synergistic changes in behavioral measures, physiological parameters, and immunoreactivity of neuropeptides in the PVT. The canonical discriminant function plots and Wilk’s lambda revealed significant separation of groups by sex and emulsifier treatment *Λ* = 0.003, *Χ*^2^(138.194) = 73.182, p < 0.001. The group centroids for Function 1 are located in opposite directions with respect to the water-treated controls.

Canonical discriminant function plot reveals the effects of each individual emulsifier treatment on each of the sexes for these two functions (Fig. 7). In males, emulsifier treatment separates along Function 1 with the group centroid for CMC treatment lower in value and the group centroid for P80 treatment greater in value than the centroid for the respective water-treated controls (Table 3). Furthermore, in females, both centroids for CMC and P80 treatment are greater in value than for water treatment. However, along Function 2, the group centroids for CMC and P80 are in the same direction with respect to the group centroids for water for both males and females. Altogether, these data demonstrate that CMC and P80 altered both physiology and behavior, with some differential effects in males and females.

**Table 3:** Functions at Group Centroids of Physiological, Behavioral, and Neuropeptidergic Effects of Emulsifier Treatment.

Sex by Treatment Label	Function				
	1	2	3	4	5
Water Male	1.601	2.495	−1.438	0.858	−0.714
CMC Male	−2.710	−2.215	−1.220	−0.663	−0.514
P80 Male	4.344	−1.401	−0.619	−0.209	0.680
Water Female	−2.813	2.266	−0.128	−0.435	0.845
CMC Female	1.374	0.694	1.992	−0.810	−0.570
P80 Female	−1.528	−1.424	1.175	1.402	0.155

Unstandardized canonical discriminant functions evaluated at group means.

## Discussion

The increased incidence of disorders related to anxiety and anti-social behavior has led to the belief that substances to which humans have been exposed as a result of industrialization might impact brain function. Such substances do not need to have direct contact with the brain. Rather, substances that impact the gut-brain axis and/or the intestinal microbiome might influence brain function and, consequently, behavior. In accord with this notion, we report here that the synthetic dietary emulsifiers CMC and P80, which have previously been shown to impact gut microbiota to induce low-grade inflammation and metabolic disorders, can also influence behavior. Specifically, we observed herein that consumption of CMC and P80 alters anxiety-like and sociability behavior. Such differences occurred in a sex-specific manner with distinct patterns of change in microbiota, neuropeptide expression, and behavior in male and female mice. Taken together, these data suggest that the sex-specific changes to microbiota composition induced by emulsifier consumption could drive the sex differences in physiological, neural, and behavioral effects of dietary emulsifiers.

Sex-specific patterns in brain and behavior were paralleled to some extent by differences in metabolism. Specifically, despite emulsifiers clearly promoting adiposity in male and female mice, an increase in overall weight was only seen in males (Supplemental Fig. 5). This discrepancy may arise because we only weighed the perigonadal (periepididymal in males; periovarian in females) white adipose tissue. While mice have several other fat depots^31,32^, the perigonadal adipose tissue pad has previously been demonstrated to respond to dietary changes^33,34^. When male and female mice are placed on calorie restriction following a high fat diet, females show a reduction predominantly in the gonadal fat pad size whereas males show a reduction in overall fat mass^34^. Nonetheless, the differences observed between the effects of emulsifier treatment on adiposity and the relative body weight in females suggest that emulsifiers induce a sex-specific change in body composition.

Sex-specific changes on spleen and colon weights following emulsifier consumption may result from sex-specific alterations of the composition of the gut microbiota following emulsifier treatment. The current study found sex differences in the microbiota of water-treated controls from both the previous study^26^ and the present one, consistent with studies demonstrating sex differences in microbiota in C57Bl/6 J mice^35^. Emulsifier treatment eliminated many of these sex differences, demonstrating the strong impact of emulsifiers on microbiota composition in yet another way. In addition, we observed sex-specific changes in microbiota composition of mice treated with emulsifiers, suggesting that some of the sex differences seen in the physiology and behavior may arise from the microbiota. While microbiota composition analysis and behavior assessment were performed after 84 and 49 days of emulsifier exposure, we previously reported that dietary emulsifier effects on the microbiota are rapid and seen *in vitro* after only few days of exposure^27^.

Although emulsifier treatment induced changes in measures of anxiety-like behavior, there is difficulty in interpreting these changes in terms of anxiety levels. For example, in males, emulsifier treatment reduced the time spent in the center portion of the open field without altering locomotive behavior or anxiety-like behaviors in the elevated plus maze, light/dark box, or marble burying test. This discrepancy between these three tests might mean that emulsifier exposure impacts passive coping or normal anxiety, for which the open field test has been suggested to be a more sensitive test than for active coping or pathological anxiety^36,37^. Moreover, the multivariate analysis showed that the aggregate of behaviors in the open field test (e.g, numbers of jumps, ambulatory episodes, circles, etc.) differed in emulsifier versus water-treated animals. In addition, emulsifier consumption increased the distance travelled in the elevated plus maze but not in the open field test. Although these effects cannot easily be interpreted in terms of changes in levels of anxiety or activity, they suggest that emulsifier treatment fundamentally impacts the organization of behavioral patterns.

Sex-specific alterations of the microbiota may have led to sex-specific changes in behavior, as found, for example, by the emulsifier-induced reduction in time spent in the center of the open field in males but not in females, or in social behavior in females but not male mice. A recent report indicates that offspring of dams fed on a high fat diet have social deficits, and that microbiota transplantation of such dysbiotic microbiota is sufficient to transfer such social deficits^6^. These data suggest that specific alterations of the gut microbiota may be critical for the behavioral effects of emulsifier treatment^6^. It is important to note that while our previous data using *in vitro* microbiota system and fecal microbiota transplantation to germfree mice demonstrated that the detrimental effects of emulsifier on metabolism are driven by direct effects on the intestinal microbiota^26,27^, we cannot yet rule out that emulsifier-effects on brain and behavior are microbiota-independent.

Effects of emulsifier treatment on weight gain may be reflected in the increases of AgRP-IR in the arcuate nucleus, the location of the AgRP-expressing neuronal cell bodies, and reductions in αMSH-IR in the PVT, an area that projects to key regions that contribute to both food intake and anxiety-like behaviors^38,39^. AgRP stimulates food intake and reduces anxiety-like behaviors, while αMSH inhibits food intake and increases anxiety-like behaviors^28–30^. Therefore, if changes in peptide levels directly correlate with changes in peptide release, the changes in AgRP-IR and αMSH-IR are consistent with the increase in food intake by emulsifiers noted in our original study^26^. Given that we did not observe a general increase in anxiety-related behaviors across tests in the current study, the relationship of the changes in AgRP-IR and αMSH-IR with anxiety-related behaviors is unclear.

Emulsifier treatment effects on behavior were not reflected in effects on the microglial population, which were not affected. It is important to note, however, that we only measured the microglial marker Iba1-IR, and therefore can not rule out that other neuroinflammatory markers, such as interleukin-6 (IL-6) or activation of the NFKB pathway, are increased following emulsifiers, as they are following high-fat diet^40^.

While determining the extent to which studies in mice are relevant to humans is inherently difficult, even in studies of metabolism where human and mice can be assayed by quite similar assays, it is especially hard to do so for behavioral disorders, whose complexity and heterogeneity make them difficult to model in mice. This caveat notwithstanding, we submit that our data support the general notion that some cases of behavioral disorders may have been impacted by exposure to modern chemical stressors and, more specifically, that synthetic dietary emulsifiers may be one such stressor. Given the ability of behavior to impact metabolic disorders, for example by impacting food consumption or energy expenditure, it is very difficult to disassociate CMC and P80’s effects on metabolism and behavior. Rather, we submit that such effects are likely intertwined, which may generally reflect the increased societal incidence of a broad range of diseases associated with inflammation. Our results thus indicate that dietary emulsifiers may be one specific perturbant of the gut-brain axis that can promote such diseases. Identification of the range of substances that can likewise perturb this axis and, subsequently, reducing exposure to such substances may be a means to staunch disease states characterized by altered behavior.

## Methods

### Animals

Six C57Bl/6 Jdams with litters (3 male and 3 female 14-day-old pups) were purchased from Charles River Laboratories. Mice were housed in ventilated transparent OptiMouse plastic cages with Bed-O-Cobs® and AlphaDri bedding (35.6 × 48.5 × 21.8 cm; at Georgia State University). Lights were set to a 14 h:10 h light:dark cycle (lights off at 0900 ET), and ambient temperature was maintained at 23 °C. Food (Purina rodent chow no. 5001) and water were available *ad libitum*. On postnatal day 21 (P21), mice were weighed and placed in a plastic container for approximately 20 minutes to collect feces for later analysis. Mice were put into a new cage such that each experimental group contained mice from all litters and that offspring from all six dams were used in all test and measures performed, except for one male in the water-treated group who developed a severe mal-occulsion and needed to be euthanized during the course of the experiment (Supplement Fig. 1). Cages were given reverse-osmosis treated Atlanta city drinking water with sodium carboxymethylcellulose (CMC; Sigma, St. Louis, MO), or with polysorbate-80 (P80; Sigma) (1% in each case), or with no additives. The drinking water and emulsifier solutions were changed weekly. Body weights were measured weekly and expressed relative to the body weight on P21. All procedures were in accordance with the Guide for Care and Use of Laboratory Animals and approved by the Animal Care and Use Committee at the Georgia State University (protocol number A15002).

### Behavioral Testing

Starting on P70, behavioral tests were conducted once a week for 6 weeks, with a week between each test (Supplemental Fig. 1). Behavioral tests were conducted in the following order: Open Field Test, Elevated Plus Maze, Light/Dark Box, Marble Burying Task, Three-Chambered Sociability Task, and Porsalt Forced Swim Test. As behavioral responses can be altered by prior test history, the tests were conducted in order of least to most invasive or disruptive^41,42^. In order to avoid shifts in the circadian rhythms of the experimental animals, behavioral testing occurred within the first 4 h after the start of the dark and active phase and was conducted under dim red light (between 20–30 lux) except for the Light/Dark box, which was illuminated by overhead lights (between 300–400 lux). Arenas were cleaned with 70% ethanol between trials. Behavioral tests were videotaped using a Sony camcorder for later analysis by AnyMaze version 4.96 (Stoelting, Co., Wood Dale, IL) or The Observer version XT11 (Noldus Information Technology Inc., Wageningen, The Netherlands). An experimenter blinded to the treatment conditions scored behavioral tests in the Observer.

#### Open Field Test

Locomotor behavior was assessed in a 43.2 × 43.2 × 30.5 cm (WxLxH) Plexiglas arena (Med Associates, Inc., St. Albans, VT) containing 2 arrays of infrared transmitters strips (16 beams each) located on the bottom of the arena (in the X and Y plane). The center zone of the arena was defined as square containing the center 8 beams (e.g., beams 4–12) in the X and Y plane. Each mouse was placed into the arena with its nose facing the wall and allowed to freely investigate for 10 min. The total distance traveled, the total time spent in the center of the arena, and circling behaviors, which are defined as movements below a preset ambulatory threshold, were calculated by Activity Monitor (Med Associates, Inc.,) on a computer connected to the open field arenas.

#### Elevated Plus Maze

An elevated plus maze with two open and two closed arms was used. Arms were 10 cm W × 50 cm L, connected by a 10 × 10 cm^2^ center chamber. Closed arms had 40 cm H walls. The maze was elevated 50 cm from the floor. Mice were placed in the center of the arena and allowed to explore for 5 min. All trials were video-recorded from a digital camera mounted above the maze and connected to a computer. The number of entries into and the total time spent in the open arms, closed arms, or center were quantified by AnyMaze.

#### Light/dark Box

A 14.5 cm W × 30 cm L × 14 cm H chamber divided into a light and dark compartment was used. The light compartment (20 cm L) was made of white acrylic, and the dark compartment (10 cm L) of opaque black acrylic and covered. An opaque insert with a 5 cm W × 5 cm H opening connected the compartments. Mice were placed in the light compartment facing away from the entry into the dark chamber and allowed to freely investigate the chamber for 5 min. All trials were video recorded from a digital camera mounted above the Light/Dark box and connected to a computer. The number of entries into the dark chamber and total time spent in the light compartment were quantified in AnyMaze.

#### Marble Burying

A Plexiglas arena (24 cm W × 46 cm L) was filled 4 cm deep with Alpha-dri bedding (Shepherd Specialty Paper, Fibercore, Cleveland, OH, USA). Mice were placed into the arena, and after a 5 min habituation period, mice were removed and twenty marbles (17 mm) were evenly spaced on top of the bedding. Mice were placed in the center of the arena and video-recorded for 10 min. The number of marbles buried, as defined by being ½ or more covered with bedding, and the latency to bury the first marble were quantified using the Observer.

#### Three Chambered Sociability

A 24 × 74 × 24 cm (L × W × H) polycarbonate apparatus was divided into three equally sized chambers with openings 9 cm W to allow free movements between compartments. At either end of the apparatus was an (9 cm W × 10 cm H) opening beside which the stimulus cages were placed. The stimulus cages were 10 cm W × 10 cm L × 10 cm H polycarbonate cage with grid (10 × 10) of small holes 0.5 cm in diameter to allow transfer of visual and olfactory cues, while limiting physical interaction to nose contact or whisking.

#### Sociability test

Following a 5 min habituation period in which the mouse was allowed to explore the entire three-chambered apparatus, the experimental animal was removed, and an unfamiliar sex- and age-matched C57Bl6/J mouse was placed inside one of the stimulus cages beside one of the side chambers. An identical stimulus cage containing a novel object was placed beside the opposite chamber. The test animal was returned to the middle chamber and allowed to freely investigate the apparatus for 10 min.

The location of the novel mouse and the novel object were alternated between left and right chambers on consecutive sessions. The time spent and the numbers of entries into each chamber were measured using AnyMaze. The time spent sniffing or actively investigating the stimulus chambers over the 10 min test was scored in The Observer. A preference score was calculated by dividing the time spent investigating the novel mouse by the total time spent investigating the novel mouse and the novel object.

#### Social Preference test

Immediately following the 10 min sociability test, the experimental mouse was removed from the three-chambered apparatus, and the novel object was replaced with an unfamiliar stimulus sex- and age- matched C57Bl6/J mouse. The original stimulus mouse used in the sociability portion of the test remained in its cage beside one chamber of the apparatus. Identical measures were scored as in the sociability test: time spent in each chamber, entries between chambers, and time spent investigating each stimulus mouse.

#### Porsolt Forced Swim Test

A vertical Plexiglas cylinder (40 cm H × 18 cm diameter) was filled with 3 L of 30 °C (±2 °C) water. Mice were placed in the center of the cylinder and video recorded for 5 mins. The latency and duration of immobility were quantified in the Observer. Immobility was defined by the absence of movement or only small movements of posterior paws that did not result in displacement of the water. At the end of the test, mice were removed from the cylinder and placed in a recovery cage on a heating pad until they were dry and then returned to their home cage.

### Euthanasia and Tissue Collections

One day following completion of behavioral testing (P105), mice were deeply anesthetized under isoflurane (5%v/v) and body weight was recorded. Blood was collected from the retrobulbar intraorbital capillary plexus. Mice were euthanized by cervical dislocation, and the colons, spleens, livers, adipose, feces, and brains were collected for subsequent analysis. Hemolysis-free serum was generated by centrifugation blood samples using serum separator tubes (Becton Dickinson, Franklin Lakes, NJ). The weight and length of the colon and weights of the spleen, liver, and perigonadal adipose fat depot were recorded and normalized to the body weight. Brains were removed and fixed in a 5% acrolein in sodium phosphate buffer (0.1 M, pH 7.4) at 4 °C, followed by cryoprotection in 30% sucrose in phosphate buffered saline (PBS: 0.05 M, ph7.4).

### Immunohistochemistry

Brains were sectioned (30 µm) in the coronal plane with a cryostat and stored in a cryoprotectant solution (ethylene glycol/sucrose in sodium phosphate buffer) until immunostained. Free-floating sections were rinsed three times in Tris-buffered saline (TBS; 0.05 M Tris, 0,9% NaCl, pH 7.6), then incubated for 30 min in 0.05 M sodium citrate in TBS. After rinsing in TBS sections were places for 30 min in 0.1 M glycine in TBS, rinsed again, and placed into blocking solution (10% normal goat serum (NGS), 0.4% Triton-X and 1% H2O2 in TBS) for 30 min. Sections were incubated overnight in one of the following primary antibodies diluted in 2% NGS and 0.4% Triton-X in TBS; anti-agouti-related peptide (AgRP; Phoenix Pharmaceuticals; 1:250000), anti-alpha-melanocyte stimulating hormone (MSH; Millipore; 1:100000), and anti-ionized calcium-binding adaptor protein (Iba1; Wako Laboratoy; 1:30000). The next day, sections were rinsed three times in TBS containing 1% NGS and 0.02% Triton-X and incubated in biotinylated secondary antiserum [goat anti-rabbit for AgRP, and Iba1 immunoreactivity; rabbit anti-sheep for MSH (Vector Laboratories, Burlingame, CA)] diluted 1:800 in TBS with 2% NGS and 0.32% Triton-X for 1 h. This was followed by rinses in TBS containing 0.4% Triton X, incubated in avidin-biotin complex (Vectastain Elite ABC Kit; Vector Laboratories) diluted to 1:800 in TBS for 1 h, followed by three TBS rinses and three sodium acetate buffer rinses. Finally, the staining was visualized using nickel-enhanced diaminobenzidine (DAB) Substrate Kit (Vector Laboratories). Sections were mounted onto gelatin-coated slides and coverslipped with Permount.

### Image Analysis

Slides were anatomically-matched and analyzed by an investigator blinded to the experimental groups. Sections were imaged using a Zeiss Axio Imager M2 microscope connected to an ORCA-R2 CCD digital camera (Hamamatsu Photonics). Gray-scale images of cell bodies, when present, and fibers immunopositive for AgRP, MSH, and AVP (Supplemental Fig. 6) were analyzed in Image J 1.43 u (National Institutes of Health, Bethesda, MD). Iba-immunoreactivity was analyzed as in^43^. The region of analysis was outlined in each section. Subjects for which the relevant sections were damaged or unavailable were dropped from a given analysis. All immunoreactivity is quantified by the mean area stained in pixels.

### Fecal microbiota 16s rRNA gene sequencing and sequences analysis

16S rRNA gene amplification and sequencing were done using the Illumina MiSeq technology following the protocol of Earth Microbiome Project with their modifications to the MOBIO PowerSoil DNA Isolation Kit procedure for extracting DNA (www.earthmicrobiome.org/emp-standard-protocols)^44,45^. Bulk DNA was extracted from feces collected on P21 and P105 using a PowerSoil-htp kit from MoBio Laboratories (Carlsbad, CA, USA) with mechanical disruption (bead-beating). The 16S rRNA genes, region V4, were PCR amplified from each sample using a composite forward primer and a reverse primer containing a unique 12-base barcode, designed using the Golay error-correcting scheme, which was used to tag PCR products from respective samples^45^. We used the forward primer 515 F 5′-*AATGATACGGCGACCACCGAGATCTACAC***TATGGTAATTGT**GTGCCAGCMGCCGCGG TAA-3′: the italicized sequence is the 5′ Illumina adapter B, the bold sequence is the primer pad, the italicized and bold sequence is the primer linker and the underlined sequence is the conserved bacterial primer 515 F. The reverse primer 806 R used was 5′-*CAAGCAGAAGACGGCATACGAGAT* XXXXXXXXXXXX **AGTCAGTCAG CC** GGACTACHVGGGTWTCTAAT-3′: the italicized sequence is the 3′ reverse complement sequence of Illumina adapter, the 12X sequence is the golay barcode, the bold sequence is the primer pad, the italicized and bold sequence is the primer linker and the underlined sequence is the conserved bacterial primer 806 R. PCR reactions consisted of Hot Master PCR mix (Quantabio, Beverly, MA, USA), 0.2 μM of each primer, 10–100 ng template, and reaction conditions were 3 min at 95 °C, followed by 30 cycles of 45 s at 95 °C, 60 s at 50 °C and 90 s at 72 °C on a Biorad thermocycler. PCRs products were purified with Ampure magnetic purification beads (Agencourt, Brea, CA, USA), and visualized by gel electrophoresis. Products were then quantified (BIOTEK Fluorescence Spectrophotometer) using Quant-iT PicoGreen dsDNA assay. A master DNA pool was generated from the purified products in equimolar ratios. The pooled products were quantified using Quant-iT PicoGreen dsDNA assay and then sequenced using an Illumina MiSeq sequencer (paired-end reads, 2 × 250 bp) at Cornell University, Ithaca.

Forward and reverse Illumina reads were joined using the fastq-join method^46,47^, sequences were demultiplexed, quality filtered using Quantitative Insights Into Microbial Ecology (QIIME, version 1.8.0) software package^48^. QIIME default parameters were used for quality filtering (reads truncated at first low-quality base and excluded if: (1) there were more than three consecutive low quality base calls (2), less than 75% of read length was consecutive high-quality base calls (3), at least one uncalled base was present (4), more than 1.5 errors were present in the bar code (5), any Phred qualities were below 20, or (6) the length was less than 75 bases). Sequences were assigned to operational taxonomic units (OTUs) using UCLUST algorithm^49^ with a 97% threshold of pairwise identity, and classified taxonomically using the Greengenes reference database 13_8^50^. A single representative sequence for each OTU was aligned and a phylogenetic tree was built using FastTree^51^. The phylogenetic tree was used for computing the unweighted UniFrac distances between samples^52,53^. rarefaction were performed and used to compare abundances of OTUs across samples. Principal coordinates analysis (PCoA) plots were used to assess the variation between experimental group (beta diversity). Alpha diversity curves were determined for all samples using the determination of the number of observed species. LEfSE (LDA Effect Size) was used to investigate bacterial members that drive differences between groups^54^. The threshold on the logarithmic LDA score for discriminative features was set to 2.0, and the alpha values for the factorial Kruskal-Wallis test and pairwise Wilcoxon test between subclasses were set to 0.05.

In addition to fecal samples collected from the animals used in this current study, the 16s sequences previously generated from^26^ were reanalyzed by combining gene sequences from both male and female mice treated with water (male: 12; female: 12), CMC (male: 11; female: 12), and P80 (male: 10; female: 9).

### Statistical analyses

Data were analyzed using IBM SPSS Statistics Version 21 (IBM) and visualized using GraphPad Prism 7.0c (GraphPad Software, La Jolla, CA). Body weights were analyzed by a repeated measure ANOVA, with sex and treatment as factors, followed by Fishers’ LSD as post hoc analyses. Anxiety-like and social behaviors were analyzed by a two-way ANOVA with treatment and sex as the factors, followed by Fishers’ LSD as post hoc analyses. Data were also analyzed by MANOVA, following by discriminant analysis in order to reveal patterns in the aggregate behavioral changes. Differences in the post-hoc comparisons were noted as significant *p < 0.05. For clustering analysis on principal coordinate plots, categories were compared and statistical significance of clustering was determined via Permanova.

## Supplementary information

Supplementary text and figures
