# Supplementary material for: Dietary emulsifiers consumption alters anxiety-like and social-related behaviors in mice in a sex-dependent manner
Source: Sci Rep. 2019 Jan 17;9:172. doi: 10.1038/s41598-018-36890-3 (PMC6336787; doi:10.1038/s41598-018-36890-3)
Supplement: Supplementary file 1 — Supplementary text and figures [file 41598_2018_36890_MOESM1_ESM.pdf]

Dietary emulsifiers consumption alters anxiety-like and social-related behaviors in mice in a sex-dependent manner

Mary K. Holder[1a], Nicole V. Peters[1], Jack Whylings[1], Christopher T. Fields[1], Andrew T. Gewirtz,[2] Benoit Chassaing[1,2, 3], and Geert J. de Vries[1, 3]

[1] Neuroscience Institute, Georgia State University, Atlanta, GA 30303, USA
[2] Institute for Biomedical Sciences, Georgia State University, Atlanta, GA 30303, USA
[3] These authors contributed equally

Corresponding authors:
Geert J. de Vries
PO Box 5030
Atlanta, GA 30302-5030
404-413-5558
Email: devries@gsu.edu

Benoit Chassaing
Georgia State University
Atlanta GA 30303
404-413-6225
Email: bchassaing@gsu.edu

[a]Current affiliation:
School of Psychology
Georgia Institute of Technology
Atlanta, GA 30332, USA

Keywords: microbiota; anxiety; sociability; carboxymethylcellulose; polysorbate 80; alpha-melanocyte stimulating hormone; agouti-related peptide

Supplemental Figure Legends

Supplemental Figure 1. Experimental Timeline. Male and female C57Bl/6 mice were weaned on post-natal day 21 (P21), started on either water control or a 1% solution of either sodium carboxymethylcellulose (CMC) or polysorbate-80 (P80). In addition, feces were collected for microbiota analysis. Behavioral testing started at P70, with one test per week in the order indicated. One day after completing the last behavioral test, animals were euthanized and feces, the brain and other organs were collected.

Supplemental Figure 2. Effects of dietary emulsifiers on microbiota. Male and female C57Bl/6 mice were exposed to drinking water containing CMC or P80 (1%). Linear discriminant analysis coupled with Effect Size (LEfSe) was of taxa that differ significantly between male and female mice within water, CCM, and P80 treatments at time of weaning, P21 and at the time of collections, P105. Phylogenetic branching that differs by treatment within male (A, B) and female (E, F) mice at P21 and within male (C, D) and female (G, H) mice at P105.

Supplemental Figure 3. Sex differences in microbiota in mice treated with dietary emulsifiers. Male and female C57Bl/6 mice were exposed to drinking water containing CMC or P80 (1%) in data previously reported in Extended Data Figure 3 in Chassaing et al., 2015. Principal coordinates analysis (PCoA) of the unweighted UniFrac distance matrix of fecal microbiota showing clustering by treatment when male and female mice are combined into a single PCoA (A). Treatment group is indicated by point color (blue, water; orange, CMC; purple, P80). PCoA of the unweighted UniFrac distance matrix of fecale microbiota also show clustering by sex in (B). Sex is indicated by point color (red, female; green, male).

Supplemental Figure 4. Additional measures of anxiety-like behaviors in mice treated with emulsifiers. Male and female C57Bl/6 mice were exposed to drinking water containing

CMC or P80 (1%) for 12 weeks and tested for anxiety-like behavior weekly starting at P70. (A) There was no effect of either emulsifier-treatment [$F_{(2, 29)}$ = 0.106, p=0.90] or sex [$F_{(1, 29)}$ = 0.59, p=0.45] on the distance traveled in the open field arena. (B) There was no effect of either emulsifier-treatment [$F_{(2, 29)}$ = 0.1995, p=0.82] or sex [$F_{(1, 29)}$ = 0.1972, p=0.66] on the time spent on the open arms. (C) The number of entries onto the open arms was not affected by either emulsifier treatment [$F_{(2, 29)}$ = 0.006, p=0.99] or sex [$F_{(1, 29)}$ = 0.05, p=0.82]. (D) There was no effect of either emulsifier-treatment [$F_{(2, 29)}$ = 0.11, p=0.90] or sex [$F_{(1, 29)}$ = 0.13, p=0.73]on the time spent in the closed arms. (E) The number of entries into the closed arms was not affected by either emulsifier treatment [$F_{(2, 29)}$ = 2.18, p=0.13] or sex [$F_{(1, 29)}$ = 3.09, p=0.09]. (F) There was no effect of either emulsifier-treatment [$F_{(2, 29)}$ = 0.07, p=0.92] or sex [$F_{(1, 29)}$ = 0.68, p=0.41] on the time spent in the light in the light/dark box. Data are represented as means + SEM (n=5-6).

Supplemental Figure 5. Sex difference in relative weight gain in mice treated with dietary emulsifiers. Male and female C57Bl/6 mice were exposed to drinking water containing CMC or P80 (1%) for 12 weeks. There was a significant interaction of time on treatment, treatment, and sex on the relative body weights in the mice over time [$F_{(24, 348)}$ = 1.863, p<0.05]. In addition post-hoc analyses indicated that 6 weeks of emulsifier consumption lead to a greater body weight in male, but not female mice (*p<0.05). Data are represented as means + SEM (n=5-6).

Supplemental Figure 6. Representative photomicrographs of Agouti-Related Peptide (AgRP) and alpha-melanocortin stimulation hormone (αMSH). Photomicrographs showing the immunoreactivity (IR) for AgRP in (A) the paraventricular nucleus of the thalamus (PVT) and (B) the arcuate nucleus (Arc). Photomicrographs showing αMSH-IR in (C) PVT and (D) Arc. Scale bar: 100μm.

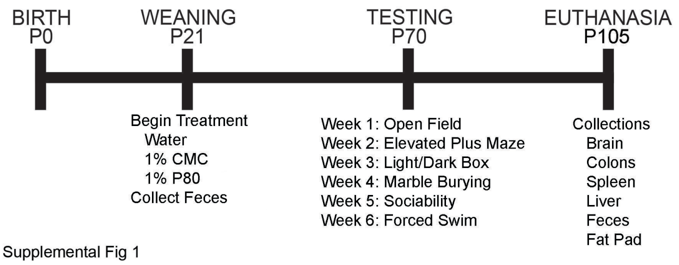

Supplemental Fig 1

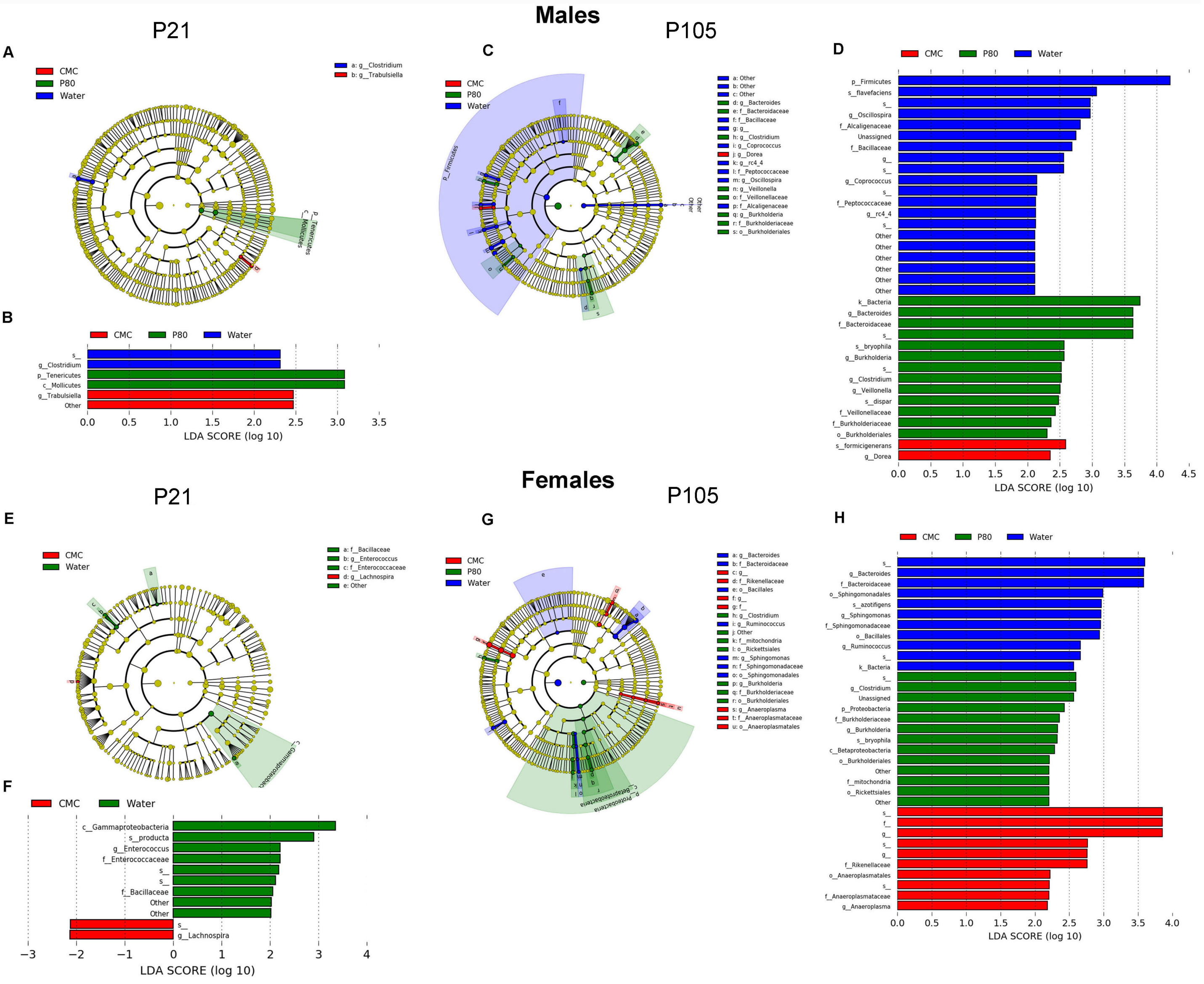

Supplemental Fig 2

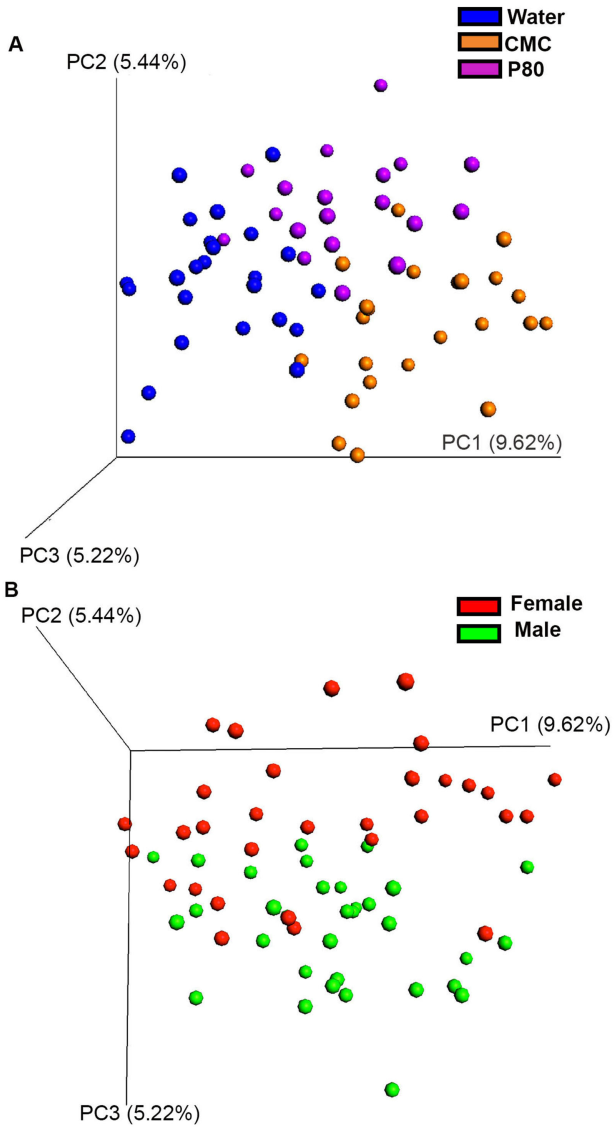

Supplemental Fig. 3

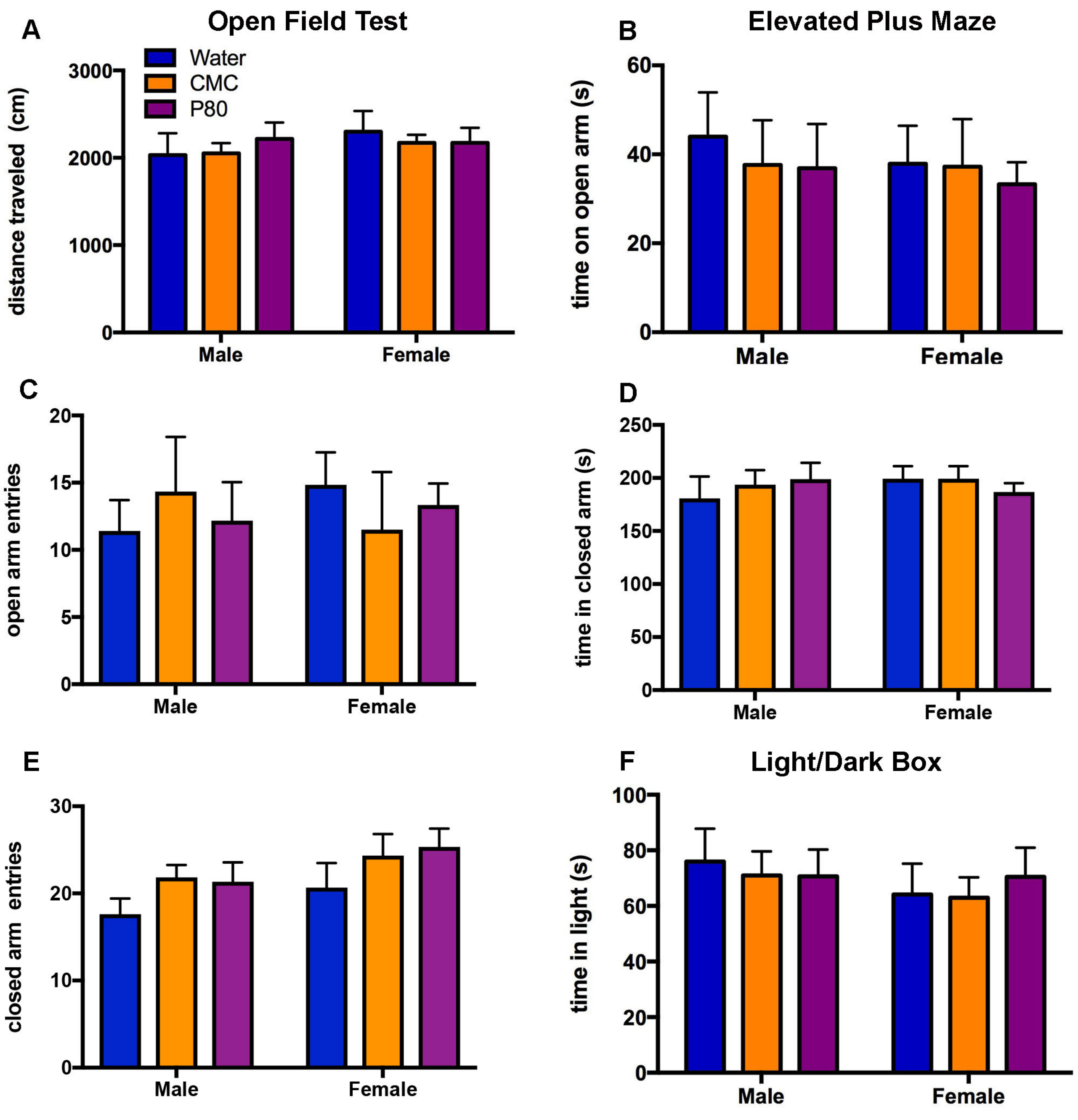

Supplemental Figure 4

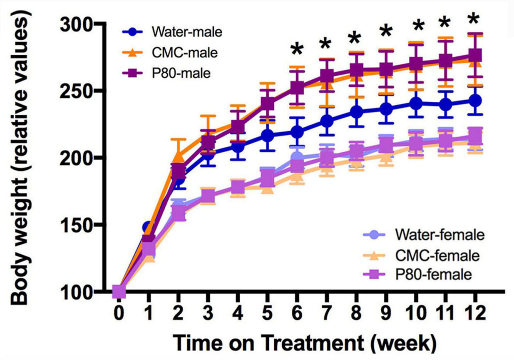

Supplemental Fig. 5

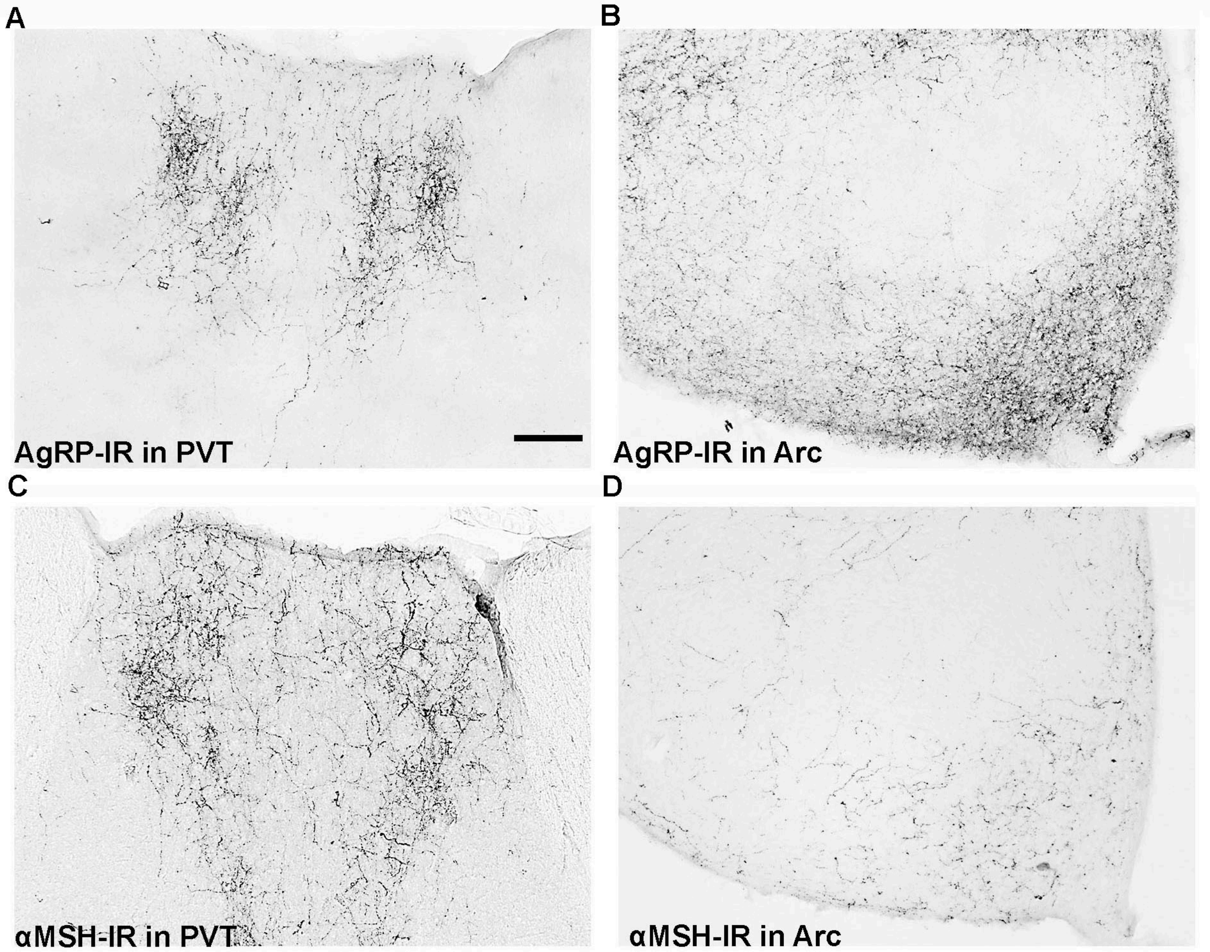

Supplemental Figure 6
